# The impact of orthopaedic research evidence on health financing in Australia

**DOI:** 10.1186/s12961-018-0314-0

**Published:** 2018-05-02

**Authors:** Martin Hua, Daniel Myers, Lachlan Host

**Affiliations:** 1 Sydney School of Public Health, University of Sydney, Sydney, NSW 2006 Australia; 2 Lyell McEwin Hospital, Adelaide, SA 5112 Australia; 3 Bathurst Base Hospital, West Bathurst, NSW 2795 Australia

**Keywords:** Evidence-based medicine, Policy making, Technology assessment, Cost effectiveness

## Abstract

**Background:**

In Australia, approval by the Medical Services Advisory Committee (MSAC) is an important step in the implementation of new health technologies. The MSAC considers health technology assessments (HTA) when submitting a recommendation to the Minister of Health on a new technology’s suitability for public funding. Despite being such a critical tool in formulating policy, there has been little scrutiny on the impact of limited evidence on the performance of a national HTA agency’s mandate. We aim to determine the proportion of HTAs of orthopaedic technologies prepared for the MSAC that were supported by higher levels of evidence for effectiveness, and whether this affected the MSAC’s ability to conclude on efficacy. We also investigated whether the availability of higher level evidence affected the performance of cost-effectiveness analyses.

**Methods:**

We performed a cohort study of all HTAs prepared for the MSAC from 1998 to 2017 with regards to new technologies in orthopaedic surgery.

**Results:**

We identified seven HTAs encompassing nine orthopaedic technologies for inclusion. Higher levels of evidence were available for assessing the technology’s effectiveness in six out of the nine technologies. The results did not show a statistically significant relationship between the availability of higher level evidence and MSAC’s ability to make a clear conclusion on the assessment of effectiveness (*P* = 0.5). The proportion of HTAs where a cost-effectiveness analysis was performed was significantly higher (*P* < 0.05) when higher levels of evidence were available for the assessment of effectiveness.

**Conclusions:**

The results indicate that there is a paucity of high quality evidence in the formulation of health policy with regards to the implementation of new orthopaedic technologies in the public healthcare system. This represents an opportunity for strong leadership from surgeons to help develop the tools needed for effective clinical decision-making.

## Background

It is incumbent upon policy-makers to use evidence-based medicine to guide decisions with regards to which medical services and procedures should be allocated government funds. This is essential to ensure that finite resources are allocated wisely to those services that are proven to offer the greatest benefit in improving healthcare. This paper intends to identify the degree to which evidence-based medicine influences this decision-making process with respect to orthopaedic techniques and procedures in Australia.

Randomised controlled trials (RCTs), when performed well, provide the gold standard in evidence for analysing efficacy of a medical intervention. Historically, difficulties have been cited in the performance of RCTs in the context of surgical interventions, including barriers to randomisation, difficulty in blinding, inherent variabilities of surgeon competency and technique, surgical learning curves, and patient’s equipoise [1]. These factors have limited the quality and quantity of randomised trials of surgical interventions. In Australia, one study noted that only 19.6% of orthopaedic procedures currently performed had at least one RCT supporting the operative treatment over non-operative alternatives [2]. Evidence shows that this has impacted on clinical decision-making on a day-to-day basis and healthcare decisions on surgical interventions are less likely to be based on RCTs [2, 3].

The Australian Institute of Health and Welfare recognises arthritis and musculoskeletal diseases as one of nine national health priority areas, accounting for a substantial amount of disease burden in Australia [4]. Orthopaedics is the surgical specialty that focuses on injuries and diseases of the body’s musculoskeletal system [5]. The Federal Government has identified potential health gains in investing in new technologies to assist Australians with orthopaedic issues, including a recent $13.3 million investment to “*fund three medical breakthroughs to help people with severe disabilities walk again and support thousands of Australians facing crippling back pain*” [6]. This is one example of government investment in the innovation and development of new health technologies. However, it is unknown to what degree decisions for the allocation of funding are based upon scientifically sound and robust evidence.

Key to the integration and delivery of a newly developed health technology are health technology assessments (HTA), which represent a multidisciplinary field of policy analysis that studies medical, social, ethical and economic implications of a new technology [7]. In Australia, the Medicare Benefits Schedule (MBS) is responsible for subsidising the cost of procedures performed in public hospitals with public funds. The MBS requires that a formal HTA is undertaken during the consideration process for public funding of new orthopaedic technologies other than prosthetic devices [8, 9]. This is to ensure the “*optimum value for money in the Government’s subsidisation of medical services, as well as prioritising the uptake of effective new technologies and procedures*” [9]. Specifically, it is the Medical Services Advisory Committee (MSAC) which advises the Minister for Health on the listing of MBS subsidies for orthopaedic technologies other than prostheses. MSAC approval, and the subsequent access to public funding, is the crucial facilitator of the uptake of the new technology in Australia and important for widespread consumer uptake [10].

In advising the Minister for Health, the MSAC Terms of Reference require it to consider “*the strength of evidence in relation to the comparative safety, effectiveness, cost-effectiveness, and total cost of the medical service*” under assessment [11]. The HTAs used by the MSAC to make recommendations on orthopaedic technologies will be dependent on the availability of the published evidence. The aforementioned scarcity of orthopaedic- and surgical-related RCTs can impact on the quality of an assessment of safety and effectiveness, and may also place restrictions on any assessment of cost-effectiveness [12]. Cost effectiveness is an important consideration as there exists a perception amongst stakeholders that financial impacts are a significant factor in attracting public funding [13]. This paper will examine the use of higher level evidence in the performance of a national HTA agency’s mandate.

Our aims were thus to (1) determine the proportion of the total HTAs performed by the MSAC, in relation to orthopaedic technologies, that were supported by higher levels of evidence (i.e. higher than level II according to the National Health and Medical Research Council guidelines), (2) determine whether the availability of higher level evidence affected MSAC’s conclusion of effectiveness, and (3) investigate whether the availability of higher level evidence limited the performance of cost-effectiveness analyses by MSAC.

## Methods

### Data source

We performed a cohort study of HTAs prepared for the MSAC from 1998 to 2017 with regards to new technologies in orthopaedic surgery. We defined ‘orthopaedic surgery’ as the application of a manual or instrumented surgical technique by a surgeon in the diagnosis or treatment of injury, disease or deformity to the body’s musculoskeletal system. HTAs are usually publicly listed and available on the MSAC website. We reviewed each HTA of orthopaedic technologies that were completed and for which information was available on the MSAC website. We excluded applications that were withdrawn and any assessments for which information was unavailable on the MSAC website.

### Data extraction

We extracted data on the general characteristics of each included HTA report, including the name of the orthopaedic technology or procedure and year of the HTA. As part of its evaluation process, the MSAC makes an assessment of the evidence presented to them in the HTA report. This is usually made against a comparator procedure, which is considered the current gold standard at the time for the same disease entity. To evaluate the quality of evidence used in the decision-making process, the authors used the National Health & Medical Research Council Guidelines 2000 [14]. Each of the included MSAC assessments were evaluated, and ascribed a level of evidence in accordance with the guidelines. The authors recorded whether the MSAC considered level I (systematic reviews) or level II (RCTs) evidence as part of its assessment. We categorised qualitative conclusions regarding the MSAC’s assessment for safety, effectiveness and cost-effectiveness. The categorisation subsets were either favourable (the orthopaedic technology was safer or more effective or cost-effective than the comparator), equivocal (the evidence supporting the orthopaedic technology was unclear) or unfavourable (the orthopaedic technology was not safer or more effective or cost-effective than the comparator). If a cost-effectiveness analysis had been performed this was also documented. Finally, the corresponding recommendation by the MSAC to support or not support Medicare funding was noted. Data was abstracted using a standardised data form.

### Outcomes and data analysis

A Fischer exact test was used to analyse the relationship between the availability of higher level evidence and the MSAC’s ability to make a clear (favourable or unfavourable) or equivocal conclusion on the assessment of effectiveness. A 2 × 2 table was used and the level of significance was set at *P* < 0.05. A Fischer exact test was also used to analyse the relationship between the availability of higher levels of evidence for the assessment of effectiveness, and any subsequent performance of a formal cost-effectiveness analysis in a 2 × 2 table. The level of significance was set at *P* < 0.05.

## Results

The MSAC received 323 applications for the performance of HTAs during the assessment period, of which 14 were identified as possibly relating to orthopaedic technologies. Of these, seven HTAs were excluded (Table 1) – two due to applications being subsequently withdrawn, three were not specifically related to orthopaedic surgical procedures performed by surgeons, one was missing on the MSAC website, and one had an incomplete assessment at the time of review. We identified seven HTAs encompassing nine orthopaedic technologies for inclusion (Table 2).

**Table 1:** Reasons for exclusion of health technology assessments from analysis

Year of assessment	Health technology	Reason for exclusion
2002	Intra-articular viscosupplementation for treatment of osteoarthritis of the knee	Intra-articular viscosupplementation for treatment of osteoarthritis of the knee Application did not relate to a procedure performed specifically by an orthopaedic surgeon
2011	Assessment of application for joint injection items	Application related specifically to rheumatologists
2012	Review of Medicare-funded wrist surgery services	Application withdrawn
2012	Matrix-induced Autologous chondrocyte implant	Application withdrawn
2014	Review of Medicare-funded finger fracture services	HTA missing on MSAC website
2015	Assessment of foot and ankle services by podiatric surgeons	Application related specifically to podiatrists
Incomplete	Vertebroplasty for severely painful osteoporotic vertebral fractures of less than 6 weeks duration	MSAC process incomplete at time of review

**Table 2:** Orthopaedic health technology assessments versus cost effectiveness and higher level evidence

Year of assessment	Orthopaedic technology	Higher evidence effectiveness	Cost effective analysis performed
2002	Intradiscal electrothermal anuloplasty	✘	✘
2006	Artificial intervertebral disc replacement (cervical)	✔	✔
2006	Artificial intervertebral disc replacement (lumbar)	✔	✔
2007	Lumbar non-fusion posterior stabilisation devices (pedicle screw device - Dynesys)	✘	✘
2007	Lumbar non-fusion posterior stabilisation devices (interspinous spacers - X STOP, Wallis, Coflex, DIAM)	✘	✘
2009	Computer-navigated total knee arthroplasty	✔	✔
2010	Matrix-induced autologous chondrocyte implantation and autologous chondrocyte implantation	✔	✔
2011	Artificial intervertebral disc replacement in patients with cervical degenerative disc disease	✔	✔
2017	Minimally invasive, lumbar decompression and dynamic stabilisation using an interlaminar device, with no rigid fixation to the vertebral pedicles, of one or two lumbar motions	✔	✘

**Fig. 1:**
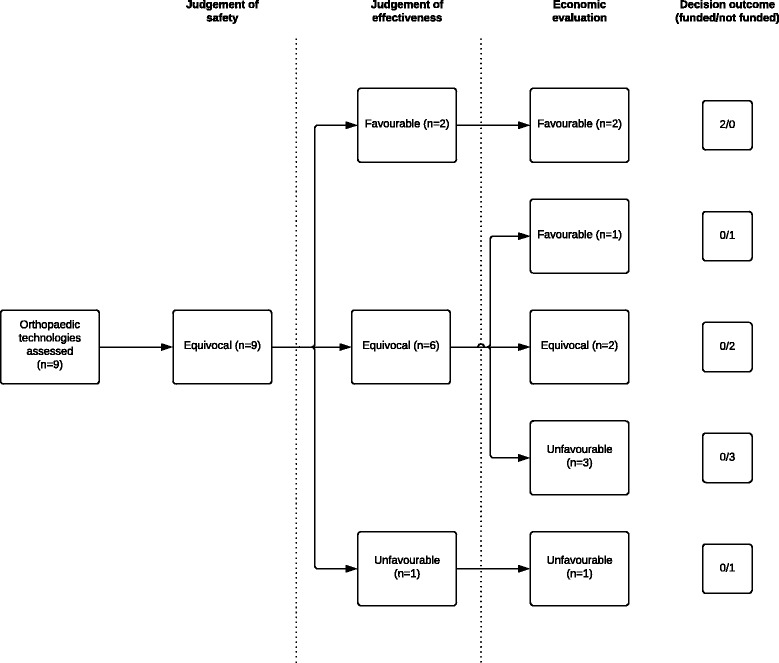
Analysis of health technology assessments

In six out of the nine technologies assessed (66.6%), higher levels of evidence were available to the MSAC when assessing the technology’s effectiveness. The assessment of effectiveness for three out of the nine technologies showed clearly favourable or unfavourable evidence (Fig. 1) and higher levels of evidence were available to MSAC for each of these assessments (Table 2). However, primary outcome results did not show a statistically significant relationship between the availability of higher level evidence and the MSAC’s ability to make a clear or equivocal conclusion on the assessment of effectiveness (*P* = 0.5).

In five of the six assessments where higher levels of evidence for the assessment of effectiveness were available, a formal cost-effective analysis was subsequently performed. In the remaining assessment (of minimally invasive lumbar decompression, application 1422), a cost-minimisation analysis was performed [15]. No cost-effectiveness analyses were performed when higher evidence was unavailable for the assessment of effectiveness. Applying Fisher’s exact test, the proportion of HTAs where a cost-effectiveness analysis was performed was significantly higher (*P* = 0.048) when higher levels of evidence were available for the assessment of effectiveness.

The MSAC made a recommendation to support MBS funding for two (25%) of the technologies assessed (Fig. 1). Higher levels of evidence were available for the assessment of effectiveness in both these technologies, with the MSAC finding the evidence favourable. Cost-effectiveness analyses were performed in both HTAs with favourable findings.

## Discussion

The Federal Government recognises that HTAs performed for the MSAC play a key role in allocating scarce healthcare resources [16]. It is well established that there is a dearth of higher level evidence in orthopaedic surgery restricting day-to-day clinical decision-making. However, until now there has been no review of the implications of limited evidence on the performance of a national HTA agency’s mandate with regards to orthopaedic surgery. This is especially pertinent in the Australian context, as Australia’s MBS subsidises healthcare costs for both public and private services [9]. MSAC approval and subsequent access to MBS funding has been viewed as important for the widespread distribution of available technologies [10]. Without MBS funding for the surgical service, its availability to the general public may be limited and clinical decision-making may thus be a moot point. In this regard, we found no statistically significant association between the use of higher level evidence in the HTA and a MSAC finding of unequivocally favourable or unfavourable evidence for effectiveness. This finding is not unique to surgical procedures, as evidenced by Merlin et al. [17], who drew similar conclusions when analysing whether evidence quality and methodology influences government decisions to publicly fund diagnostic medical tests; however, they noted that evidence may have contributed to increased decision-maker certainty with regards to funding decisions.

Nevertheless, the findings regarding orthopaedic procedures presented herein should be viewed in the following context. Each of the three orthopaedic technologies for which MSAC found clearly favourable or unfavourable evidence for effectiveness were supported by level II or higher evidence. Conversely, for the three technologies for which higher levels of evidence for effectiveness were not available, each was found to have equivocal evidence for effectiveness. These points would, prima facie, suggest that the MSAC requires higher level evidence to effectively exercise evidence-based judgments for orthopaedic technologies. However, the results were affected by the inclusion of three HTAs (assessment of ‘Artificial intervertebral disc replacement (cervical)’, ‘Minimally invasive lumbar decompression and dynamic stabilisation using interlaminar device’, and ‘Computer-navigated total knee arthroplasty’ [18, 19]), for which RCTs were available for the assessment of effectiveness but which resulted in an equivocal finding by the MSAC. In each of these HTAs, the MSAC cited specific issues with the quality of the RCT available. In the assessment of ‘Artificial intervertebral disc replacement (cervical)’, the MSAC noted that “*the trial enrolled few participants, did not report full data, had short-term follow-up (24 months), and participants, investigators and outcome assessors were not blinded to treatment*” [18]. With regards to the assessment of ‘Minimally invasive, lumbar decompression and dynamic stabilisation using interlaminar device’, the MSAC noted that, amongst other concerns, the results of the RCT “*lacked transparency, and was subject to a high risk of bias*” [15]. For the assessment of ‘Computer-navigated total knee arthroplasty’, the MSAC noted that trial outcomes focused on radiological improvements, but that it was “*unclear whether the significant improvements in radiological outcomes translate to measurable clinical benefits for the patient, such as a reduction in revision rates*” [19]. Nonetheless, each of the new orthopaedic procedures that eventually received recommendation for public funding was supported by at least level II ‘favourable’ evidence for effectiveness, perhaps reaffirming that the MSAC requires higher level evidence to effectively exercise its mandate.

Brauer et al. [12] previously found that a lack of higher level evidence in orthopaedic surgery limited the performance of high-quality cost analyses and, where performed, cost analyses were of lower quality than in other specialties. Our results, which we believe are the first to consider this issue with regards to a national HTA agency and to orthopaedic technologies, supports Brauer et al.’s conclusion. In particular, no cost-effectiveness analyses were performed when higher evidence was unavailable for the assessment of effectiveness. In some of these instances, a cost-minimisation analysis was performed instead of a cost-effectiveness analysis. However, Dakin and Wordsworth contended that cost-minimisation analyses may bias measures of uncertainty, causing overestimation or underestimation of the probability that a treatment is cost-effective [20]; a cost-effectiveness analysis would thus be preferred to avoid bias. There was one instance in which a HTA included higher evidence for the assessment of effectiveness, but a cost-minimisation analysis was performed instead of a cost-effectiveness analysis; this was for the assessment of ‘Minimally invasive, lumbar decompression and dynamic stabilisation using an interlaminar device’, and as previously noted, there were concerns that results from the RCT used were subject to a high risk of bias [15]. Collectively, the use of cost-minimisation analysis in the absence of higher evidence for the assessment of effectiveness, or where concerns are raised about the risk of bias from such evidence, may further reinforce Brauer et al.’s [12] conclusion. This is particularly pertinent as global increases in surgical expenditure have led to uncertainty about the marginal benefit of new surgical technologies. In the 2014/2015 Federal Budget [21], the Federal Government emphasised cost-effectiveness as a barometer for sustainable healthcare expenditure. In this regard, it was found in a previous analysis that the MSAC was more likely to make a recommendation against Medicare funding where the evidence of cost-effectiveness was equivocal or unfavourable [22]. As noted previously, funding may have a strong impact on a technology’s ultimate dissemination. In the context of the limited availability of high quality cost-analyses, this raises the possibility of type II errors. That is, the implication that a funding decision can be affected by the outcome of a limited economic evaluation, if performed at all, may prompt concerns that access is denied to procedures that may otherwise be an efficient use of societal healthcare resources.

We have limited the current analysis to orthopaedic surgery given the historic limitations cited in the gathering of higher evidence in surgery and specifically the documented impact of these limitations on clinical decision-making in Australia. Given the findings, it is recommended that a future analysis consider this issue across all surgical subspecialties. Furthermore, in this instance, we did not consider the impact of RCTs on the assessment of safety. This is because the Therapeutic Goods Administration generally assesses the safety of new medical technologies as a precursor to the MSAC’s HTA process [23].

A potential selection bias exists whereby, since 2010, companies applying for MSAC approval for a new technology may elect to have a submission-based assessment and not permit the full reports to be publicly published. Hence, while the outcome of the MSAC’s determination is evident, the quality of the evidence used to draw that conclusion is not always so. It should also be recognised that the sample size for the study was small, totalling nine HTAs. This is most likely due to the relatively low number of non-prosthetic-related orthopaedic surgical procedures being produced. This relatively low number may mean that the addition of a few more HTAs, either as they are submitted to the MASC over time, or through the application of different search criteria may change the results of the study.

Finally, a decade ago, Rogers issued a ‘call to arms’ for surgeons to move towards research on “*patient- and society-oriented and risk-adjusted outcomes, including cost-effectiveness, quality of life, and functional outcomes*” [24]. However, Hohman et al. [25] recently found that Australian-trained surgeons or surgical trainees accounted for only a minority of publications originating from Australia in the 15 highest-ranking orthopaedic journals.

## Conclusion

The study results did not show a statistically significant relationship between the availability of higher level evidence and the ability of policy-makers to make a clear or equivocal conclusion on the assessment of effectiveness of new technologies in Australia. The realities of modern healthcare are such that policy-makers may, at times from necessity, drive a cost-effectiveness agenda at a trade off against patient choices and patient care. As society has become accustomed to having access to any new intervention, this may perpetuate a real or perceived conflict amongst surgeons, patients and policy-makers. As such, clinicians and policy-makers must remain mindful of the importance of including high quality, scientific evidence in funding decisions and maintain an interest in developing the tools needed for effective clinical decision-making at both micro and macro levels.

## Abbreviations

HTA: Health Technology Assessment
MBS: Medicare Benefits Schedule
MSAC: Medical Services Advisory Committee
RCT: randomised controlled trial
